# Impact of COVID-19 on Pediatric Inflammatory Bowel Diseases—From Expectations to Reality

**DOI:** 10.3390/jpm14040399

**Published:** 2024-04-09

**Authors:** Laura Mihaela Trandafir, Elena Lia Spoiala, Gabriela Ghiga, Nicoleta Gimiga, Paula-Diana Budescu, Vasile Valeriu Lupu, Lacramioara Butnariu, Elena Cojocaru, Gabriela Paduraru

**Affiliations:** 1 Faculty of Medicine, “Grigore T. Popa” University of Medicine and Pharmacy, 16 University Street, 700115 Iasi, Romania; laura.trandafir@umfiasi.ro (L.M.T.); elena-lia.spoiala@umfiasi.ro (E.L.S.); vasile.lupu@umfiasi.ro (V.V.L.); paduraru.gabriela@umfiasi.ro (G.P.); 2 Saint Mary Children Hospital, Vasile Lupu Street, no 62-64, 700309 Iasi, Romania; budescu.paula-diana@email.umfiasi.ro; 3 Department of Medical Genetics, Faculty of Medicine, “Grigore T. Popa” University of Medicine and Pharmacy, 16 University Street, 700115 Iasi, Romania; ionela.butnariu@umfiasi.ro; 4 Morpho-Functional Sciences II Department, “Grigore T. Popa” University of Medicine and Pharmacy, 16 University Street, 700115 Iasi, Romania; elena.cojocaruu@umfiasi.ro

**Keywords:** inflammatory bowel disease, COVID-19, children

## Abstract

Viral infections have always been considered a threat to global health, with numerous outbreaks across time. Despite the relative recent experience with coronavirus-associated diseases such as severe acute respiratory syndrome (SARS) and Middle East respiratory syndrome (MERS), severe acute respiratory syndrome-2’s (SARS-CoV-2) continuous evolution displays a different behavior. With a tropism for both respiratory and digestive mucosa, coronavirus disease 2019 (COVID-19) and inflammatory bowel disease (IBD) seem to share a particular common background. Current literature offers evidence that viral alteration of the immune system, inflammatory intestinal tissue damage, increased intestinal permeability, incomplete viral clearance with viral antigen persistence, and intestinal dysbiosis, might explain SARS-CoV-2–IBD relationship in terms of etiopathogenesis and evolution. The hyperinflammatory state that both entities have in common explains the lack of success of current IBD therapy, raising the need for new personalized therapeutic options, with better outcomes for IBD and COVID-19 as well. This review aims to summarize the current available data on pediatric IBD evolution, management, and outcomes in the post-COVID period, with an emphasis on the particular aspects of the SARS-CoV-2–IBD relationship in children.

## 1. Introduction

Coronaviruses are not a novelty for humanity. In the last 20 years, severe acute respiratory syndrome (SARS) and Middle East respiratory syndrome (MERS), coronavirus-associated diseases, have raised awareness of their potential threat, with outbreaks all over the world. Opening a new page in history, the coronavirus disease 2019 (COVID-19) pandemic has put enormous pressure on healthcare systems all around the world, affecting the quality of both acute and chronic medical care. Almost four years ago, medical attention was focused on the management of severe acute respiratory syndrome-coronavirus 2 (SARS-CoV-2) infection in terms of acute features. As the years went by, the novel coronavirus showed its complex nature and generated interest in studying its long-term complications and interactions with other pathologies [1,2,3,4,5].

It is a well-known fact that the respiratory tract is primarily affected by SARS-CoV-2 infection. Yet, gastrointestinal (GI) involvement is also common. Although children have a less severe presentation than adults [6], there is evidence that 15–84% of pediatric patients with COVID-19 experience at least one gastrointestinal symptom, including abdominal pain, diarrhea, or vomiting [7]. Several physiopathological mechanisms, including an altered barrier function of the intestinal wall, local inflammation, and SARS-CoV-2-associated-intestinal dysbiosis, are cited as related to these GI manifestations [8].

Inflammatory bowel diseases (IBD), including Crohn’s disease and ulcerative colitis, are chronic conditions characterized by inflammation of the gastrointestinal tract. The prevalence of IBD has increased globally, and new information is being uncovered from regions where it was previously undocumented [9]. Approximately 10% of all cases involve the onset of the disease in children, associating a severe and chronic inflammatory evolution that requires lifelong therapy, a significant financial burden, and the continued assistance of the healthcare system [10].

Both characterized by a disrupted inflammatory response and a pro-inflammatory background, COVID-19 and IBD display a particular connection in terms of disease onset and evolution. Although the understanding of this relationship is still evolving, several studies have shed light on the potential impact of COVID-19 on children with pre-existing IBD as well as on the onset of new pediatric IBD cases [7,8,9,10].

In the past few years, the COVID-19 pandemic has posed unique challenges for children with IBD. Preliminary studies suggest that children with IBD may not be at a higher risk of contracting COVID-19 compared to the general population [11]. However, those with severe active IBD, moderate to severe malnutrition, or high-dosed systemic corticosteroids have an increased risk for severe disease [12]. A study in children and young adults with IBD receiving infliximab or vedolizumab showed a significantly lower and less durable antibody response to natural infection compared with adult non-IBD patients [13].

Current literature offers a serious body of information related to adult patients’ risk of COVID-19, appropriate management, and virus-related outcomes [14,15]. However, there is a lack of knowledge about these matters in the pediatric population. While the influence of COVID-19 on children with IBD is still being studied, there is evidence to suggest that the virus may have an important influence on the course and management of IBD [11,16]. This review aims to summarize the current available data on pediatric IBD evolution, management, and outcomes in the post-COVID period, with an emphasis on the particular aspects of SARS-CoV-2–IBD relationship in children. In the era of personalized medicine, there is an urgent need for an individualized, targeted therapy to improve the long-term prognosis as well as the quality of life of children with chronic diseases, as traditional IBD therapy might not be enough for patients in the context of a SARS-CoV-2 infection.

## 2. The Impact of COVID-19 on the Incidence, Outcomes and Management of IBD

During the COVID-19 pandemic, there was growing concern regarding the impact of the virus on immunosuppressed individuals, including children with IBD [11,17,18]. Did the reduced exposure to environmental triggers and improved hygiene practices reduce the incidence of IBD, or did the stress and the cytokine storm associated with COVID-19 lead to disease flares or new-onset IBD cases? Table 1 depicts the main studies reporting the influence of COVID-19 on IBD evolution in children.

The pandemic has posed challenges in terms of access to healthcare services for children with IBD. The disruptions in routine medical care, including clinic visits, endoscopies, and laboratory tests, may lead to delays in diagnosis, treatment adjustments, and monitoring of disease activity, as families with children with GI complaints might have avoided hospitals for fear of contracting the virus [21]. Telemedicine has emerged as a valuable tool to bridge this gap and ensure ongoing care for children with IBD during these challenging times [27].

One aspect of concern during the pandemic was the potential for COVID-19 to exacerbate the symptoms of IBD in children. The use of immunosuppressive medications, such as corticosteroids or biologic agents, which are commonly prescribed to control inflammation in IBD, has been linked to a higher risk of bacterial and viral infections [28]. In addition, as psychological stress was considered a promoting or relapsing factor for IBD in both children and adults [29], it has been hypothesized that the pandemic can trigger flare-ups or worsen the existing symptoms of IBD. Moreover, viral persistence within the gastrointestinal tract may contribute to chronic inflammation and subsequent autoimmune reactions [30]. As a result, concerns have been raised regarding the potential for increased incidence and severity of COVID-19 infection among individuals with IBD.

However, current literature offers no certain evidence that COVID-19 can influence the course of IBD in this manner. IBD can impact individuals of all ages. The relationship between IBD and SARS-CoV-2 has garnered attention in the scientific community, particularly in understanding how these conditions interact in different age groups. Both children and adults with gastrointestinal (GI) diseases, including celiac disease and IBD, do not appear to have an increased risk of experiencing severe cases of COVID-19, including hospitalization, critical care requirements, and mortality [31,32]. Compared to adults, however, children with IBD have a milder COVID-19 disease course, and fewer of them require hospitalization or intensive care for COVID-19 [12].

Moreover, the treatment for IBD may influence the outcome of COVID-19: Both adults and children on systemic corticosteroids or 5-aminosalicylates are more susceptible to experiencing severe cases of COVID-19 [12,33]. In a cross-sectional survey, the medication adherence rate in pediatric patients with IBD during the pandemic was similar to the adherence rate among adults with IBD [21]. Furthermore, Bezzio et al. [32] reported that the rate of disease flare among patients with IBD, children, and adults in remission was not significantly different between those with SARS-CoV-2 infection (n = 28 of 118, 23.7%) and those without viral infection (n = 25 of 137, 18.3%) (*p* = 0.35) [32].

Nonetheless, we must take into consideration multisystem inflammatory syndrome in children (MIS-C) as a possible SARS-CoV-2 complication that could occur in IBD patients. According to the Centers for Disease Control and Prevention (CDC), MIS-C represents a rare, but potentially life-threatening condition that usually occurs 2–6 weeks after a child is infected with SARS-CoV-2, due to an aberrant immunological response associated with fever, multi-system organ dysfunction, and an increase in inflammatory biomarkers [34]. Due to the actual epidemiological context, practitioners should be aware that an acute symptomatology in an IBD patient might suggest a disease flare-up as well as a concurrent MIS-C. Even though the clinical features of MIS-C are very heterogeneous, gastrointestinal involvement is often seen in pediatric patients [35]. This might be secondary to the richness of angiotensin-converting enzyme 2 (ACE2) receptors and the increased expression of transmembrane serine protease 2 (TMPRSS2) found in the gastrointestinal tract that allows SARS-CoV-2 to directly injure the intestinal tissue and to induce inflammation as well [10,35].

In their case series, Krawiec et al. [36] described the clinical course and therapeutic management of four pediatric patients previously diagnosed with IBD, with overlapping disease flare-up and MIS-C. All patients were under anti-inflammatory treatment for IBD, and one of them received vaccination against COVID-19. Compared to a normal IBD flare, all of them presented severe symptomatology requiring aminosalicylic acid (ASA) and intravenous immunoglobulin (IVIG) treatment. Furthermore, the weak response to therapy in three patients imposed intravenous corticosteroid administration, associating a favorable outcome [36]. Severe symptomatology increased serologic inflammation markers, a particular aspect on endoscopic and histologic examination, in association with a poor response to conventional IBD therapy was also remarked by other authors, as a constellation that is suggestive for MIS-C occurrence [37,38,39,40].

With regards to the SARS-CoV-2 vaccination impact on the IBD pediatric population, we must mention the controversy that dominated the situation. As soon as the rumors of the existence of an anti-SARS-CoV-2 vaccine appeared, concern was raised regarding their safety and efficiency, making parents anxious and prone to refuse to vaccinate their children [41]. However, vaccination was reported to be safe for both adults and children known to have IBD, with a low rate of vaccination-induced disease flare and a reduced risk of thrombotic events [23,42,43,44]. Studies revealed that a third vaccine dose might be able to reduce COVID-19 incidence and lower the rates of severe outcomes, including hospital admission [45,46]. Schell and colleagues reported that IBD patients on biologics are susceptible to reinfection because of their weakened IgG response to natural SARS-CoV-2 infection [33]. Furthermore, one-third of the patients under anti-TNF alpha therapy, failed to maintain high post-vaccination anti-SARS-CoV-2 IgG/IgM levels [42,47,48]. However, following vaccination, the antibody response is much higher than to natural infection, offering additional protection against a possible SARS-CoV-2 infection [13]. There is not enough data to establish if anti-SARS-CoV-2 vaccines in children lower the severity of COVID-19 or decrease the risk of triggering de novo IBD post-viral infection, but there is evidence that pediatric IBD incidence did not increase during the last period. Nonetheless, in order to decrease the risk of disease breakthrough and its potential complications, setting vaccination as a priority for this particular group becomes essential.

## 3. COVID-19—A Trigger for IBD in Children?

IBD pathogenesis consists of an altered immune response against intestinal bacterial components of the microbiota in genetically susceptible human beings. Several environmental factors that can directly affect the gastrointestinal tract were proposed as triggers for the self-sustained inflammatory process that characterizes IBD. Current literature offers evidence of a possible connection between IBD progression and intestinal infection-associated dysbiosis. Molecular mimicry might be the cornerstone of de novo IBD post-viral infection, as the immune cross-reaction of host antigens and viral epitopes promotes an altered immune response in the intestinal lining [49].

### 3.1. Insights from Molecular Biology

Given the current body of knowledge regarding the influence of viral infections on the onset of IBD, it can be assumed that any virus, including the recently identified SARS-CoV-2, may serve as a catalyst for the onset of IBD in individuals who are genetically predisposed to develop the illness or who are at risk of contracting it [11]. Because they can interfere with immune system activation and generate an excessive cytokine response, viral infections can trigger or accelerate the development of IBD [50].

The spike glycoprotein, which adheres to the extracellular domain of angiotensin-converting enzyme 2 (ACE2) on host cells, is thought to be the primary cause of SARS-CoV-2 infection [51,52]. As ACE2 and viral spikes become linked, ACE2’s physiological function decreases because of its reduced expression and activity [53]. The epithelial cells of the respiratory tract exhibit high amounts of ACE2, but the infection can spread to several organs from there since this protein is expressed in various other organs. Furthermore, significant inflammation and cytokine activation brought on by an intensified immune response in certain individuals might result in multiple organ failure and severe breathing problems. The brush boundary membrane of small intestine enterocytes, particularly proximal and distal enterocytes, has been shown to express ACE2 at high levels [54]. Furthermore, by cleaving the virus’s spike glycoprotein on the cell membrane, the existence of cellular serine proteases, transmembrane protease serine 2 (TMPRSS2) and transmembrane protease serine 4 (TMPRSS4), enhances SARS-CoV-2 infection of enterocytes [55].

The colon and ileum contain high levels of ACE2 and TMPRSS2, transmembrane protease, and serine 2, which are necessary for the activation of the viral S-protein peplomer [56]. As a consequence, the virus may penetrate the cells of the intestinal epithelium, sometimes aggravating any underlying intestinal tract infections or initiating new inflammation [56]. A case of acute hemorrhagic colitis induced by a gastrointestinal infection caused by SARS-CoV-2 was reported by Carvalho A et al., in relation to a 71-year-old woman. The endoscopy helped rule out other illness etiologies and verified colonic damage [57]. In a case reported by Liu Q et al., following the dissection of a patient with COVID-19, it was seen that the small intestine had segmental dilatation and stenosis and that plasma cells and lymphocytes had infiltrated the lamina propria, rectum, and duodenum [58].

An additional process is dependent on the activation of the immune system [59]. Numerous inflammatory mediators and chemokines, including interleukin (IL)-2, IL-7, granulocyte colony-stimulating factor, interferon-γ inducible protein 10, monocyte chemoattractant protein 1, macrophage inflammatory protein 1-α, and tumor necrosis factor-alpha (TNF-α), are released by SARS-CoV-2-infected cells [60]. This “cytokine storm” encourages the GI system’s immune cells to proliferate at abnormal levels, creating the premises of IBD development [60]. There is evidence that in adult patients with COVID-19, the underlying layers of the gastrointestinal tract present an important interstitial edema and contain a significant count of lymphocytes and infiltrating plasma cells [61]. Moreover, the severity of the illness and the development of various organ insufficiencies outside the lung, liver, and pancreas are correlated with the overproduction of cytokines [62].

Intestinal cells are susceptible to both direct cytopathic insults and indirect immune-mediated damage caused by SARS-CoV-2. Furthermore, the novel coronavirus can compromise the intestinal wall’s integrity by altering the expression of tight junction proteins [63]. This breach in the barrier function may trigger an immune response, leading to chronic inflammation and the development of IBD [64]. Yonker et al., found that the presence of SARS-CoV-2 in the gastrointestinal tract leads to the release of zonulin, a protein responsible for intestinal permeability regulation. When found at increased levels, it alters tight junction gene expression and increases intestinal permeability [65]. This allows SARS-CoV-2 antigens to enter the bloodstream, triggering hyperinflammation. Zonulin, however, increases its levels in conditions associated with severe inflammation, as usually seen in MIS-C [66,67]. In response, larazotide, a zonulin antagonist, reduces inflammation markers and shows comparable clinical outcomes to standard MIS-C treatments [65].

It has been almost 90 years since Crohn first postulated the idea of an infectious agent triggering IBD onset. Bacteria, viruses, parasites, they all have been proposed as relevant to IBD development. The common path would be that these pathogens determine a persistent immune dysregulation that could result in chronic GI inflammation and IBD onset in genetically susceptible individuals [68,69,70]. Although the association between SARS-CoV-2 and IBD is not fully defined, we share the idea that SARS-CoV-2 in particular has a propensity to instigate IBD compared to other infections. Viral alteration of the immune system, inflammatory intestinal tissue damage, increased intestinal permeability, incomplete viral clearance with viral antigen persistence, and intestinal dysbiosis might unravel some of the multifaceted roles that SARS-CoV-2 play in IBD etiopathogenesis and evolution.

### 3.2. COVID-19 and Gut Microbiota Alterations

The gut microbiota plays a crucial role in maintaining intestinal homeostasis and immune function [71]. COVID-19 infection has been associated with alterations in the gut microbiota composition [72]. Dysbiosis, characterized by an imbalance in the microbial community, may disrupt the delicate balance between the immune system and gut microbiota, potentially leading to the development of IBD [73].

Even when just the respiratory mucosa is affected, SARS-CoV-2 may change the gut microbial communities’ composition [73,74]. As a result of increased inflammatory mediators, the lung becomes more permeable, allowing the virus and inflammatory mediators to move from the bloodstream into the gut [62]. The greatest amount of ACE2 receptors is found in the brush border of the intestinal epithelial cells, opening the path for SARS-CoV-2 at the digestive tract level. New virions are created in the cytoplasm of GI cells upon viral entrance, and these virions are subsequently discharged into the GI tract, where they directly impair enterocyte function and cause viral shedding in the stool [73,74]. SARS-CoV-2 causes the destruction of absorptive enterocytes, which disrupts the integrity of the intestinal barrier, increasing intestinal permeability and allowing the translocation of bacteria and other antigens from the gut into the bloodstream, promoting systemic inflammation [75].

In a case-control study conducted by Nashed et al. [76], the comparison of the microbiomes of 595 young children aged 0–2 years old with asymptomatic SARS-CoV-2 infection revealed a lower presence of *Bifidobacterium bifidum* and *Akkermansia muciniphila* in affected children, both of which are known to have a protective effect against inflammation [77,78]. Proteobacteria, known to be overrepresented in children with IBD [79], was also reported to be highly expressed in children with MIS-C (11%) compared to healthy controls (5%) [80]. In symptomatic children, Romani et al. reported a decrease in overall α-diversity in the “mild” and “moderate” groups compared to the “asymptomatic” group [81]. Therefore, symptomatic COVID-19 cases were correlated with decreases in microbiome diversity, and even asymptomatic cases were associated with a decreased abundance of anti-inflammatory bacteria [75,81].

According to a recent study on the plasma microbiome of patients with SARS-CoV-2 infection, lipopolysaccharide, fatty acid-binding protein 2, and peptidoglycan, (markers of gut permeability) were found to have increased serum levels in COVID-19 patients than in controls, indicating a fragile condition of the intestinal barrier during the infection [82].

Using the AT1/ACE2 transport system, tryptophan, an essential amino acid, cannot enter the gut if SARS-CoV-2 is present at the intestinal level [83]. Along with its metabolite nicotinamide, tryptophan plays a critical role in regulating the inflammatory tendency by controlling the release of antimicrobial peptides that impact the composition of the gut microbiota [83,84]. Consequently, a disturbance in the tryptophan supply leads to a reduction in antimicrobial peptide levels and alterations in the gut microbiota, which in turn causes inflammation [83]. The molecular mechanism behind this process has just been demonstrated in mice [83,84]. Alterations in the tryptophan metabolites of intestinal bacteria resulting from altered gut microbiota in ACE2 knockout mice may also have an impact on the modulation of immunological responses through Aryl hydrocarbon receptor (AhR) activation [84]. However, data suggests that certain individuals with COVID-19, in particular, have dysbiosis of the gut microbiota due to a reduction in *Lactobacillus* and *Bifidobacterium* numbers [85].

COVID-19-associated dysbiosis is characterized by a reduction in the number of beneficial microbial strains such as butyrate-producing bacterial species, known for their anti-inflammatory properties [86]. Moreover, potential pathogenic bacterial strains raise their levels, increasing intestinal permeability and promoting inflammation, creating the premises for IBD development.

## 4. De Novo Pediatric IBD Post COVID-19

As previously mentioned, SARS-CoV-2 displays the potential to trigger IBD flares as well as the development of new IBD cases. Several studies sustain the hypothesis that immunological tolerance is lost after SARS-CoV-2 infection, secondary to dysregulated interferon production and cytokine activation that leads to an aberrant immunological response in the gut, raising the risk of intestinal injury and inflammation as seen in IBD patients [87,88].

Besides the retrospective analyses on the impact of COVID-19 on the course of IBD, there are several studies reporting newly diagnosed cases of IBD following COVID-19, both in children and adults. For instance, Kim et al. [89] reported two cases of Crohn’s disease in previously healthy male patients aged 17 and 11 years old. Swatski et al. reported a case series of 2 female patients aged 16 years old and a female patient aged 12-years old with new-onset ulcerative colitis following a recent diagnosis of COVID-19 [90]. Although these cases seem to be rare, it is important to be aware of the need for further investigations into IBD in children with persistent hematochezia, diarrhea, and/or abdominal pain following COVID-19 [91,92].

In short, de novo IBD post-COVID-19 in the pediatric population seems to be characterized by mild to moderate disease onset in terms of clinical features, inflammatory serological markers, endoscopic, and histology findings, as synthesized in Table 2. With similar findings, several case reports were found in adults as well [93,94,95,96]. Although most of the new IBD adult cases respond to traditional IBD therapy, some of the pediatric patients do not present a favorable response to oral prednisone and mesalamine, requiring anti-TNF-α agents such as infliximab. This observation suggests that in some individuals, SARS-CoV-2 immune dysregulation and its associated cytokine production might persist in elevated parameters, despite passing over the acute phase of COVID-19. On a long-term basis, these patients might require close monitoring, as this pro-inflammatory state could persist through time and cause additional complications. COVID-19 vaccination status does not seem to have an influence on disease onset or evolution [89,90]. In the adult population, however, there are new cases of IBD with onset reported after a previous SARS-CoV-2 infection, most of them with severe evolution, reflecting another aspect of the intricate SARS-CoV-2–IBD relationship [97,98,99,100].

## 5. Current and Future Perspectives on Therapeutic Approach of COVID-19-Related Pediatric IBD

Current pediatric practice uses drugs such as corticosteroids and 5-aminosalicylates in addition to immunosuppressants and biological therapy in order to achieve and preserve remission of inflammation during disease flares. ASA, steroids, oral budesonide, methotrexate, thiopurines, as well as anti-TNF therapy, anti-IL12/23, anti-integrin, and JAK inhibitors are drugs used for treating IBD, including de novo cases post-COVID-19, depending on its degree of severity [85].

However, given the presence of a “cytokine storm” and its associated hyperinflammatory state, IBD flare-ups concurrent with MIS-C seem to respond better to the MIS-C therapeutic approach than to IBD traditional drugs [37,38,39,40].

Glucocorticoids and other immunomodulatory medications, such as IVIG, represent the first line of MIS-C therapy strategies. A more aggressive immunomodulatory treatment option, including anakinra, tocilizumab, and infliximab, is taken into consideration if the patient becomes insensitive to IVIG and glucocorticoids [36]. Several pediatric case reports describe a refractory response in IBD flare and MIS-C cases, with a positive response to infliximab [37,38,39,40]. Infliximab administration was reported to ameliorate clinical symptomatology and to reduce inflammation levels, normalizing TNF-α, Il-6, and IL-8 levels [37].

## 6. Conclusions

In our opinion, the potential connection between COVID-19 and the development or exacerbation of IBD in children is a concerning and complex issue that requires further investigation. The immune dysregulation, gut microbiota alterations, viral persistence, and disruption of intestinal barrier function observed in COVID-19 infection may contribute to the development of IBD in susceptible individuals and trigger flares in individuals already known to have IBD. Due to the possible overlap between severe IBD flares and MIS-C, we believe that healthcare providers should be vigilant and prepared for atypical presentations. Moreover, investigating the potential long-term effects of COVID-19 on children with IBD, including the impact on disease progression, treatment response, and overall health outcomes, may provide valuable insights into the management of pediatric IBD and inform future treatment strategies for this vulnerable population.

## Figures and Tables

**Table 1:** Main studies reporting the relationship between COVID-19 and IBD in children.

Study, Publication Year, Country	Study Design	Population Size	Population Age (Male:Female)	Type of Inflammatory Disease	COVID-19 Cases	Relevant Findings
Arrigo et al., 2021, Italy [19]	Multicenter, retrospective, cohort study	2291	14.3 ± 1.6 years old (1296:995)	984 (42.9%) CD 1177 (51.3%) UC 130 (5.7%) IBD-U	6 cases (0.2%): -5/6 with mild infection, without requiring hospitalization -1/6 (an 18-year-old girl with UC) requiring 1 week hospitalization due to pneumonia	↓ Hospital admissions [604/2291 (26.3%) vs. 1281/2291 (55.9%); *p* < 0.001]. ↓ Hospitalizations for new diagnosis (from n = 44 to n = 27) ↓ Endoscopic re-evaluations (from n = 46 to n = 8). No changes in relapses and surgical procedures. No changes in biologic infusions.
Rosenbaum et al., 2023, New York [20]	Multicenter, retrospective, cohort study	587	14.0 (2–21) years old (333:253)	399 (68.0%) CD 159 (27.1%) UC 29 (4.9%) IBD-U	8 cases with mild course	Statistically significant increases in CD and UC cases in 2020–2022 period compared to 2016–2020.
Dorfman et al., 2021, Israel [21]	Cross-sectional telephonic survey	244	15.3 (12.6–17.1) years old (117:127)	170 (69.7%) CD 67 (27.5%) UC 7 (2.8%) IBD-U	Not mentioned	↑ Concerns regarding the attendance of regular clinics (116, 47.5%) and emergency room in case of IBD exacerbation (178, 73%). 7/244 (2.9%) patients changed or discontinued their IBD treatment due to COVID-19.
D’Arcangelo et al., 2021, Italy [22]	Retrospective, observational, single-center, cohort study	185	11 ± 3.5 years old (107:78)	101 (55%) CD 82 (44%) UC 2 (1%) IBD-U	4 cases with mild/asymptomatic course	No worsening of IBD symptoms during COVID-19 disease. No interruption in biological therapy.
Bosa et al., 2022, Italy [23]	Prospective, observational, single-center study	84	14 (1–18) years old	Not mentioned.	12 cases with mild/asymptomatic course (9 with CD, 1 with UC and 2 with IBD-U)	No worsening of IBD symptoms during COVID-19 disease. No interruption in biological therapy.
Koletzko et al., 2021, Germany [24]	Cohort, questionnaire-based study	90	6–20 years old (51:39)	44 CD 34 UC 10 IBD-U	Not clearly mentioned	Medication changes in 4.6% cases (dose reduction, change in interval, pausing or omitting ongoing or newly proposed medication). Cancelled or postponed endoscopies or surgery in only 3.8% and 0.4%, respectively.
Sansotta et al., 2021, Italy [25]	Cohort, questionnaire-based study	290	15.2 (2–18) years old	117 (40%) CD 155 (54%) UC 18 (6%) IBD-U	2 cases with mild course	No new IBD cases. Only 1 IBD flare and 1 with infectious colitis, both requiring hospital admission due to fever and gastrointestinal symptoms. No interruption in biological therapy.
Magalhães et al., 2022, Portugal [26]	Retrospective, single center study	268	15 (7–18) years old (10:5)	18 (75%) CD 6 (25%) UC	11 with mild course	No gastrointestinal complaints. No reports of complications or hospitalizations due to COVID-19. No interruption of treatment in 90% cases (3 patients interrupted treatment due to mandatory quarantine).

IBD—inflammatory bowel disease, CD—Crohn’s disease, UC—ulcerative colitis, IBD-U—inflammatory bowel disease unclassified. Arrow down signifies decreased; arrow up signifies increased.

**Table 2:** New cases of IBD in children following COVID-19.

Study, Publication Year, Country	IBD Type	Patient Age, Sex	COVID-19 Vaccination Status	Symptoms at Initial Presentation	Initial COVID-19 Treatment	Latency Period	Symptoms after Latency Period	Endoscopic Findings	Histopathological Findings	Treatment and Outcome of IBD
Kim et al., 2023, South Korea [89]	CD	17 years old, male	Not mentioned	Fever, sore throat, cough	Antipyretics	2 weeks	Nausea, vomiting, diarrhea, abdominal, pain, fever, weight loss	Small ulcers in the distal esophagus, Edematous mucosa with multiple erosions, prominent lymphoid follicles in the terminal ileum A small ulcer in the ascending colon	Chronic esophagitis with focal detached fibrinosuppurative exudate. Chronic granulomatous inflammation with a few multinucleated giant cells in the terminal ileum	Before confirmation: methylprednisolone, then prednisolone After confirmation: azathioprine + prednisolone in tapering dose No details about outcome
	CD	11 years old, male	Not mentioned	Fever	No treatment required	A few weeks	Fever, abdominal pain	Ileocecal valve deformity with mucosal edema and ulcerations. A large ulcer with cobblestone appearance in the cecum.	Focal aphthous ulcer formation with acute and chronic inflammation, and inflamed granulation tissue formation in the colon.	Before confirmation: intravenous antibiotics. After confirmation: azathioprine + prednisolone in tapering dose No details about outcome.
Morita et al., 2023, Japan [91]	UC (severe)	13 years old, female	2 Pfizer-BioNTech COVID-19 vaccines	Fever, cough, dysgeusia	No treatment required	4 weeks	Abdominal pain Diarrhea Hematochezia	Complete obliteration of the vascular pattern of the colon with erosions and some luminal bleeding	A crypt abscess and crypt distortion.	5-aminosalicylate without response, then salazosulfapyridine which was also ineffective. Prednisone with good response.
Preziosi et al., 2022, United States of America [92]	UC	10 years old, female	Unvaccinated	Diarrhea, hematochezia, fever, cough	Not mentioned	No latency period	Not applicable (no latency period)	Not mentioned	Not mentioned	Iron supplements Resolution of hematochezia and diarrhea within 2 weeks.
	UC	9 years old, female	Unvaccinated	Diarrhea, hematochezia, fever, cough	Not mentioned	No latency period	Not applicable (no latency period)	Pancolitis extending from rectum to ascending colon with normal cecum, terminal ileum.	Moderately active pancolitis with features of chronicity.	Oral prednisone, mesalamine, then Infliximab due to repeated relapses as the prednisone dose was decreased.
Swatski et al., 2023, United States of America [90]	UC (severe)	16 years old, female	2 Pfizer-BioNTech COVID-19 vaccines	Not mentioned, but no hospitalization required	No treatment required	3 weeks	Abdominal pain Hematochezia	Diffuse severe inflammation (adherent blood, altered vascularity, edema, erosions, erythema, confluent ulcerations) in the colon	Diffuse chronic active colitis with normal terminal ileum.	Oral prednisone, mesalamine, and infliximab with good response.
	UC (severe)	16 years old, female	2 Pfizer-BioNTech COVID-19 vaccines	Not mentioned, but no hospitalization required	No treatment required	8 weeks	Abdominal pain Hematochezia Headache Nausea Fatigue	Inflammation in a continuous and circumferential pattern from the anus to the cecum	Chronic active pancolitis with cryptitis from the rectum through the cecum with normal terminal ileum.	Oral prednisone, mesalamine, then escalated to infliximab due to poor response. Vancomycin for *Clostridium difficile* infection in day 40 after diagnosis.
	UC (severe)	12 years old, female	Unvaccinated	Not mentioned, but no hospitalization required	No treatment required	3 weeks	Abdominal pain Hematochezia	Diffuse moderate colitis with loss of vascularity and mild shallow ulcers throughout the entire colon.	Chronic active pancolitis throughout the colon with normal terminal ileum.	Oral prednisone, then infliximab due to worsening hematochezia.

IBD—inflammatory bowel disease, CD—Crohn’s disease, UC—ulcerative colitis.

## Data Availability

Data used in the study will be available from the corresponding authors upon request.
